# Ambient Air Pollution and Risk for Ischemic Stroke: A Short-Term Exposure Assessment in South China

**DOI:** 10.3390/ijerph14091091

**Published:** 2017-09-20

**Authors:** Pi Guo, Yulin Wang, Wenru Feng, Jiagang Wu, Chuanxi Fu, Hai Deng, Jun Huang, Li Wang, Murui Zheng, Huazhang Liu

**Affiliations:** 1 Department of Preventive Medicine, Shantou University Medical College, Shantou 515041, China; pguo@stu.edu.cn (P.G.); wangli3740@126.com (L.W.); 2 Guangzhou Center for Disease Control and Prevention, Guangzhou 510440, China; wangyl1858@sina.com (Y.W.); 13711694617@163.com (W.F.); wjg0608@126.com (J.W.); fuchuanxi@gmail.com (C.F.); 3 Department of Cardiology, Guangdong Cardiovascular Institute, Guangzhou 510100, China; doctordh@hotmail.com (H.D.); huangjungdci@163.com (J.H.)

**Keywords:** air pollution, environmental exposure, ischemic stroke, short-term, time-series model

## Abstract

Data on the association between air pollution and risk of ischemic stroke in China are still limited. This study aimed to investigate the association between short-term exposure to ambient air pollution and risk of ischemic strokes in Guangzhou, the most densely-populated city in south China, using a large-scale multicenter database of stroke hospital admissions. Daily counts of ischemic stroke admissions over the study years 2013–2015 were obtained from the Guangzhou Cardiovascular and Cerebrovascular Disease Event Surveillance System. Daily particulate matter <2.5 μm in diameter (PM_2.5_), sulfur dioxide (SO_2_), nitrogen dioxide (NO_2_), ozone (O_3_), and meteorological data were collected. The associations between air pollutants and hospital admissions for stroke were examined using relative risks (RRs) and their corresponding 95% confidence intervals (CIs) based on time-series Poisson regression models, adjusting for temperature, public holiday, day of week, and temporal trends in stroke. Ischemic stroke admissions increased from 27,532 to 35,279 through 2013 to 2015, increasing by 28.14%. Parameter estimates for NO_2_ exposure were robust regardless of the model used. The association between same-day NO_2_ (RR = 1.0509, 95% CI: 1.0353–1.0668) exposure and stroke risk was significant when accounting for other air pollutants, day of the week, public holidays, temperature, and temporal trends in stroke events. Overall, we observed a borderline significant association between NO_2_ exposure modeled as an averaged lag effect and ischemic stroke risk. This study provides data on air pollution exposures and stroke risk, and contributes to better planning of clinical services and emergency contingency response for stroke.

## 1. Introduction

Increased risk of cardiovascular disease, including stroke, linked to the exposure to ambient concentrations of air pollutants has been documented increasingly in recent years [1,2,3,4,5]. The effects of the exposure to different air pollutants on the morbidity and mortality of cardiovascular diseases have been presented [2,6,7,8].

Globally, stroke is the second leading cause of death [9], and also one of the main causes of hospitalization, long-term disability, and high medical cost [10]. The overall population burden of stroke is huge and increasing, particularly in the developing world. A recent study in the US observed associations between ischemic stroke risk and particulate matter <2.5 μm in diameter (PM_2.5_) and ozone (O_3_) exposure, suggesting future research on ambient air pollution and stroke is warranted [11].

In China, a study in 2005 reported that stroke mortality per 100,000 persons annually was 116.63 in urban areas and 111.74 in rural settings, suggesting that stroke was the second cause of death in both urban and rural settings in the country [12]. More recently, a study from Beijing city in north China showed a positive correlation between short-term PM_2.5_ exposure and hospital admissions for stroke on warm days [1]. Several previous studies have also focused the effects of elevated gaseous air pollutants including sulfur dioxide (SO_2_), nitrogen dioxide (NO_2_), and O_3_ on the emergency hospital admissions for diseases such as hypertension and pneumonia in China [13,14]. A study from Taiwan province demonstrated a significant association between the increase in NO_2_ and admissions for both primary intracerebral hemorrhage and ischemic stroke [15]. Also, the exposure to air pollutant NO_2_ increasing the risk of ischemic stroke mortality in Shanghai, China had been displayed [16]. However, there is still a lack of evidence for a connection between ambient air pollution and stroke risk in south China where the compositions of atmospheric pollutants are different from those in north China.

In particular, a recent study showed that in Guangzhou, the most densely-populated city in south China with a population of around 12.7 million in 2010, the burden of stroke mortality was relatively high in the country [17]. According to our knowledge, the association between ambient air pollution and stroke risk, and the relative disease burden in Guangzhou is still unclear.

Therefore, in order to assess the effects of the exposure to the concentrations of air pollution on the risk of stroke in south China, this present study used a large-scale multicenter database of stroke hospital admissions in Guangzhou, and analyzed the associations between ischemic stroke risk and the pollutants including PM_2.5_, O_3_, SO_2_, and NO_2_.

## 2. Materials and Methods

### 2.1. Study Area and Population

**Figure 1:**
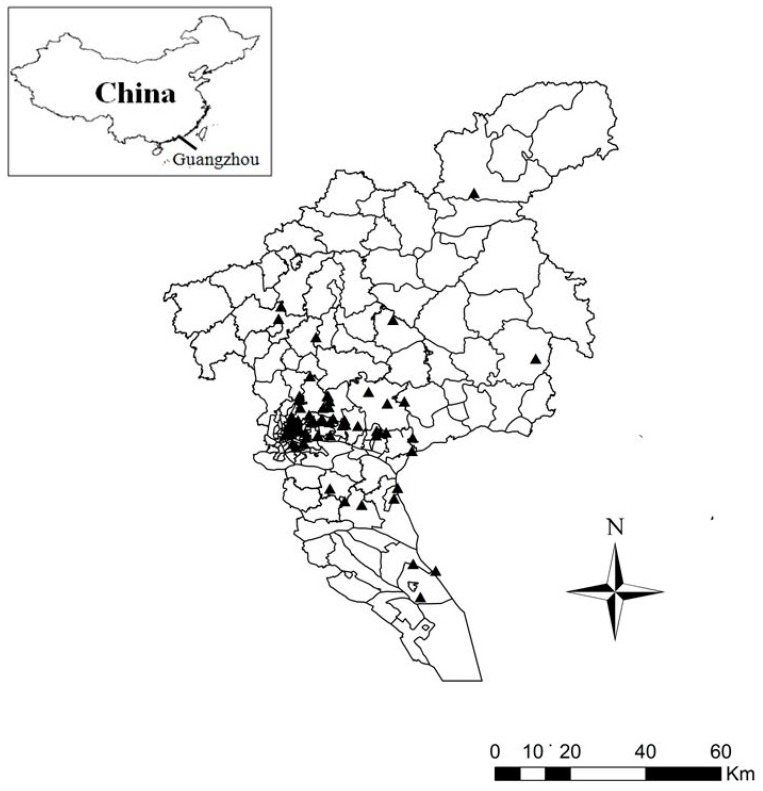
Geographical distribution of sentinel hospitals for stroke events monitoring in Guangzhou during the time period of 2013–2015. Located on the Pearl River Delta Area, Guangzhou is the capital and largest urban setting of Guangdong province, south China.

The study population was local residents who were registered in the Household Register of Guangzhou. Guangzhou is the capital and largest city of Guangdong Province, south China. As the most populous city in south China, Guangzhou is located on the Pearl River Delta Region, with a population of around 12.7 million in 2010. Hospital admission data for ischemic stroke used in this study were obtained from the Guangzhou Cardio- and Cerebrovascular Disease Event Surveillance System, which was built in 2011 by the Guangzhou Health Bureau to continuously monitor stroke and other cardiovascular events locally. At present, a total of 67 sentinel hospitals as monitoring sites of stroke events were included in the surveillance network. The sentinel hospitals from the districts including Yuexiu, Liwan, Tianhe, Haizhu, Huangpu, Baiyun, Panyu, Conghua, Huadu, Nansha, and Luogang are widely distributed in Guangzhou (Figure 1), and the study participants were permanent residents living in the study areas. Population data of these districts in Guangzhou were publicly available from the Guangzhou Statistics Information website (http://www.gzstats.gov.cn/).

### 2.2. Ischemic Stroke Case Identification

Daily admission data for ischemic stroke during the time period of 2013–2015 were abstracted by trained staff members of Guangzhou Centers for Disease Control (CDC) using a standard operation procedure. Cases identified from the surveillance system were interviewed face-to-face by trained staff of Guangzhou CDC to collect information on the demographics and relevant information. Recorded demographic information, medical history, and clinical information on stroke symptoms were obtained. If there were recurrent strokes occurring within 28 days of a previous admission, they were treated as a single event of stroke [18]. All the strokes were coded under the World Health Organization’s International Classification of Diseases, the 10th version (ICD-10) from I60 to I64, and the subgroup of ischemic strokes corresponds to the code of I63 [18].

A sequence of systematic quality control of data was performed during data management. Data entry was done by trained staff in a private medical service unit. A double entry strategy was carried out to input data to reduce errors such as mismatches and out-of-range values. Two independent investigators inspected all data of stroke admission to ensure that patients who transferred between hospitals were treated as only one admission. When detecting any missing data, we tracked and re-entered the incomplete information.

### 2.3. Air Pollutant and Meteorological Data

Daily air pollution data on PM_2.5_, O_3_, SO_2_, and NO_2_ were publicly available from the Qingyue Open Environmental Data (QOED) Center (https://wat.epmap.org/). The center is managed by the Environmental Protection Bureau of Minhang district of Shanghai, China. It aims to promote the nationwide environmental information available in public for academic research. At present, huge amounts of high-quality environmental data in relation to air and water quality are provided. Accordingly, the air pollution data in the QOED center are mainly from the source of real-time air quality monitoring data released by the government. In addition, the process of data calculation and management was according to the guidelines of China Technical Specification for Environmental Air Quality Assessment (Trial).

Historical air pollutant data for the study time period were obtained from the database. Data on PM_2.5_, SO_2_, and NO_2_ was measured in micrograms per cubic meter (μg/m^3^) and summarized as the hourly average per 24-h time period. O_3_ data was measured in micrograms per cubic meter (μg/m^3^) and summarized as the hourly maximum per 24-h time period. Of the 1095 days in the study period, there were missing values of air pollution data for the first 17 days (1.55%) in the year 2013. The small amount of missing values for each pollutant was estimated using series means, respectively.

Historical meteorological data over the study period of 2013–2015 were publicly obtained from the China Meteorological Data Sharing System (http://cdc.nmic.cn/home.do). Daily mean ambient temperature representing the average exposure of temperature throughout the 24-h period was measured in degrees Celsius, and used to account for the potential confounding effects of temperature in the models established.

### 2.4. Statistical Analysis

Descriptive analysis was used to study the basic characteristics including gender, age, ethnic group, occupation, and marital status of ischemic stroke patients by calendar year. Daily counts of stroke and daily levels of air pollutants (PM_2.5_, O_3_, SO_2_, and NO_2_) were examined individually with time series plots.

To examine the associations between the exposure to the pollutants and ischemic stroke risk, Poisson regression models were used in this study. Semiparametric regression models were established with daily counts of stroke as the dependent variable. Parametric effects in the model included day of the week modeled as indicator variables with Monday as the referent and holiday modeled as indicator variables to adjust for the effects of public holidays. Time in days was modeled using a natural cubic spline with three degrees of freedom to account for temporal trends in stroke hospital admissions over the study period. A small number of degrees of freedom were adopted because a strong seasonal trend in the time series of stroke hospital admissions was not found. Same day average ambient temperature was modeled using a natural cubic spline with three degrees of freedom to adjust for the potential confounding effects of temperature. The settings of parameters were also proposed by Lisabeth et al. in a previous study [11]. Each pollutant was considered to be separately modeled as lagged variables up to five days prior to the date of stroke occurrence as the effects on stroke may be delayed. For example, for PM_2.5_, the lag 1 model represented PM_2.5_ concentrations for the 24 h prior to the day of stroke occurrence.

We explored the potential confounding effects of one single-pollutant such as O_3_ on the association between the other pollutant such as PM_2.5_ and stroke risk given PM_2.5_ has been linked to stroke. First, we considered the independent association between PM_2.5_ and stroke risk in a single-pollutant model. Then, we added O_3_ to the model and built a new model including the two pollutants. We determined the degree to which O_3_ confounded the association between the exposure to PM_2.5_ and stroke risk. Potential confounding effects of other pollutants were assessed using this approach, and a series of two-pollutant models were established.

In addition, we constructed a multi-pollutant model simultaneously including all the pollutants in a single model to investigate the independent associations between air pollutants and stroke risk, and determined their individual magnitude of increasing stroke risk. The models were adjusted for day of the week, public holidays, temperature, and temporal trends as described above. An unconstrained distributed lag Poisson regression model was constructed to estimate the marginal effect on risk of stroke admissions associated with a unit increase in a five-day weighted average of each pollutant [19]. The marginal effect was calculated by averaging coefficients of each pollutant lags 0–5 in the model [11].

The Poisson regression models were estimated without using an offset term, reflecting the assumption that the population at risk was relatively constant over the time period [11]. The effects of pollutants in the model were summarized as relative risk (RR) or percent change for an interquartile range (IQR) increase in the pollutant levels, and standard errors were used to compute 95% confidence intervals (CI) for the estimated effects. To examine the adequacy of the models, we used an autocorrelation function (ACF) and histogram plots to check if the residuals of the models were independent and randomly distributed over time. All analyses were completed within the software of R version 3.2.2. All statistical tests were two-sided, and a *p* < 0.05 was considered to be statistically significant.

## 3. Results

Basic characteristics of stroke patients by calendar year are displayed in Table 1. There were a total of 95,562 ischemic stroke cases over the study years. The number of the ischemic stroke cases increased from 27,532 to 35,279. Based on the population data from the Guangzhou Statistics Information website, the estimated hospital admission rate for ischemic stroke in the study districts was 231.85, 272.68, and 284.95 per 100,000 in 2013, 2014, and 2015, respectively, showing an upward trend during the study period. The stroke patients in 2013, 2014, and 2015 had an average age of 71.2, 71.3, and 71.9, respectively. The number of stroke cases among males was beyond that among females, regardless of what year it was. Most of the cases occurred among participants aged 75 above years old in relation to other age groups, and around 99 percent were Han Chinese. Among the study participants, most of them were married. On average, for each ischemic stroke patient, the duration of hospital stays was 11 days, and the total hospitalization expenses slightly increased during the three-year period.

**Figure 2:**
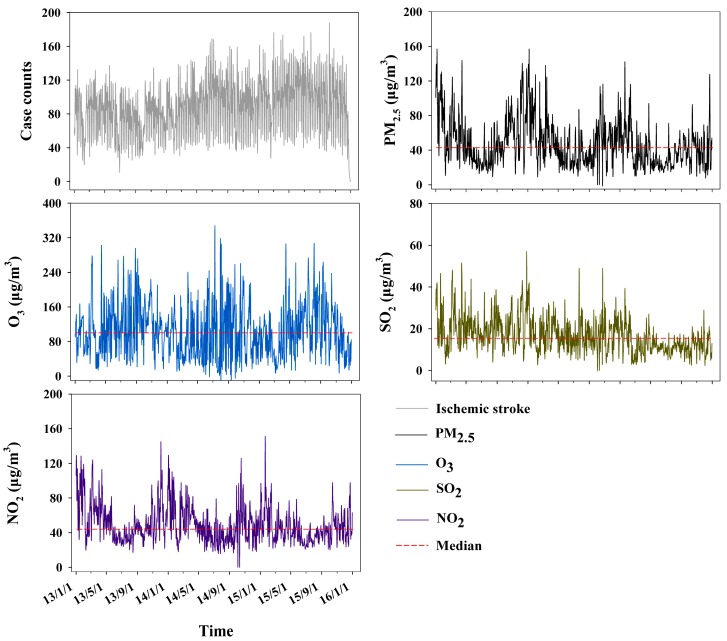
Daily number of ischemic stroke cases, and daily values of air pollutants including PM_2.5_, O_3_, SO_2_, and NO_2_ over the study time period of 2013–2015.

Daily number of ischemic stroke cases and daily levels of PM_2.5_, O_3_, SO_2_, and NO_2_ during the study period is shown in Figure 2. Median number of stroke cases per day was 89 (IQR, 63 to 108). Median level of PM_2.5_, O_3_, SO_2_, and NO_2_ over the years was 41.0 μg/m^3^ (IQR, 27.0 to 60.0), 99.0 μg/m^3^ (IQR, 59.0 to 141.0), 15.0 μg/m^3^ (IQR, 11.0 to 21.0), 44.0 μg/m^3^ (IQR, 34.0 to 60.0), respectively.

Stroke risk ratios associated with an IQR increase in air pollution concentrations are shown in Table 2. In the single-pollutant models, the effects of same-day exposure to PM_2.5_ (RR = 1.0272, 95% CI: 1.0177–1.0368), O_3_ (RR = 1.0173, 95% CI: 1.0078–1.0269), SO_2_ (RR = 1.0344, 95% CI: 1.0253–1.0435), and NO_2_ (RR = 1.0423, 95% CI: 1.0327–1.0520) on hospital admissions for stroke were suggested. The similar pattern was also observed for previous-day exposure to the pollutants. In the two-pollutant models, parameter estimates for same-day exposure to SO_2_ and NO_2_ were largely unchanged. However, the estimated effects of same-day PM_2.5_ and O_3_ exposure in the models turned out to be statistically insignificant when controlling for the effects of SO_2_ and NO_2_. In the multi-pollutant model, the effects of same-day SO_2_ (RR = 1.0179, 95% CI: 1.0054–1.0306) and NO_2_ (RR = 1.0419, 95% CI: 1.0265–1.0576) exposure on stroke risk retained statistically significance when adjusting for other pollutants in the model. Similar effects of previous-day NO_2_ and O_3_ exposures on stroke risk were also observed.

**Figure 3:**
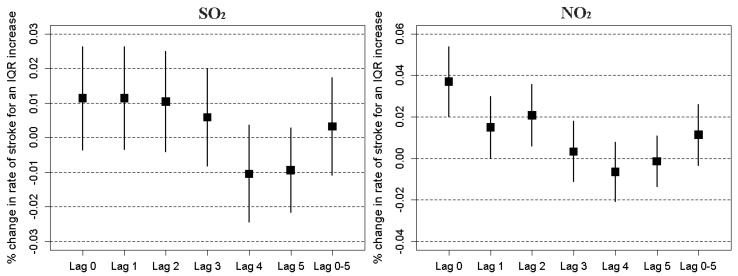
Percent change in ischemic stroke risk associated with an inter quartile range (IQR) increase in the level of NO_2_ (IQR = 26 μg/m^3^) and SO_2_ (IQR = 10 μg/m^3^), respectively.

**Figure 4:**
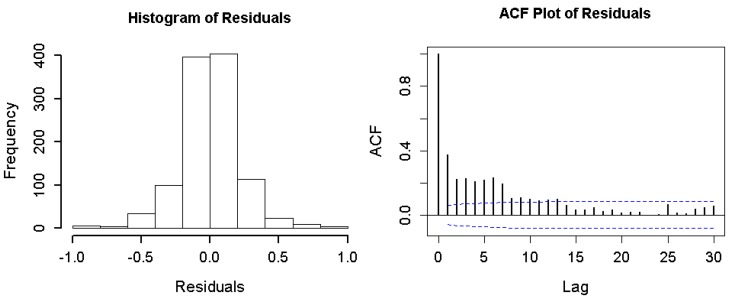
Plots of histogram and autocorrelation function (ACF) of the residuals from the constructed model to check the validity of the multi-pollutant model. The blue lines represent the confidence interval lines with a 95% coverage probability.

Figure 3 displays percent change in ischemic stroke risk associated with an IQR increase in the levels of NO_2_ and SO_2_, respectively. The results were estimated according to the constructed multi-pollutant model. The significant effects of same-day exposure to NO_2_ and SO_2_ on the increased risk of hospital admissions for stroke were observed. The individual effect was diminishing over time for each of the two pollutants. Overall, NO_2_ and SO_2_ modeled as an averaged lag effect was statistically significant, respectively. The ACF and histogram plots of the residuals from the multi-pollutant model revealed that there was not any autocorrelation in the residuals and the model had captured the patterns in the data quite well (Figure 4).

## 4. Discussion

This is one of the few studies of daily ischemic stroke hospital admissions reported so far, and is one of the few studies on short-term effects of ambient air pollutants (PM_2.5_, O_3_, SO_2_, and NO_2_) on hospital admissions based on a large-scale multicenter registry database for stroke events in China. A total of 95,562 attack admissions for ischemic stroke in 67 surveillance points in Guangzhou during 2013–2015 were included in this study, and a thorough evaluation of the effects was provided. As industrial production and traffic within Guangzhou, one of the most densely populated cities in south China, continue to increase, more people are suffering from shortness of breath, coughing, dizziness and related diseases. We observed significant associations between same-day NO_2_ and SO_2_ exposures and attack risk for ischemic stroke accounting for ambient temperature, day of the week, public holidays and temporal trends in stroke events. The results were not confounded by other air pollutants including PM_2.5_ and O_3_. Similar associations between previous-day NO_2_ and O_3_ exposures and stroke risk were also observed. The findings contribute to the literature as there is currently a scarcity of data on the association of air pollution and daily stroke rate in Guangzhou. Although this study found that the magnitude of elevated risk of stroke due to NO_2_ and SO_2_ exposures in Guangzhou was relatively small, the potentially harmful impact on public health due to the exposure in the area cannot be understate.

We found that the estimated hospital admission rate for ischemic stroke in the study districts enjoyed an upward trend over the study time period. Most of the stroke cases occurred among participants aged over 75 years old in the study population, and the risk of hospital admissions for ischemic stroke seemed to be higher in males. The finding was consistent with that of several previous studies [20,21]. However, on average, the stroke patients in our study area had longer duration of hospital stays than the residents of the Minnesota metropolitan area in the USA [22].

The evidence of the effect of PM_2.5_ exposure on stroke risk is still limited in China. A recent study from Beijing in north China demonstrated the elevated risk of hospital admissions for ischemic and hemorrhagic stroke on warm days due to the short-term PM_2.5_ exposure [13]. However, when adjusting for other air pollutants in our models, parameter estimates for PM_2.5_ were largely changed, and the magnitude of elevated risk of stroke hospital admissions due to PM_2.5_ exposure turned out to be statistically insignificant. According to Guo et al. [13], median level of PM_2.5_ over the years of 2013–2014 in Beijing was 71.4 μg/m^3^ (IQR, 37.0 to 119.0), which was remarkably higher than the Figure 2 41.0 μg/m^3^ (IQR, 27.0 to 60.0) in Guangzhou in our work. This might partly explain the observed difference. Since people living in Beijing had greater PM_2.5_ exposure, they seemed to have a higher risk of developing ischemic stroke. Therefore, continuous studies based on enlarged sample size should be performed to evaluate the association in Guangzhou in the future. Also, we observed a statistically insignificant association between same-day O_3_ exposure and stroke risk. This result was supported by a previous study in which O_3_ demonstrated a similar association with stroke risk in the Nueces County of Texas, America [11].

For the constructed multi-pollutant model, the same-day exposure to NO_2_ remained significantly associated with hospital admissions for ischemic stroke when the pollutants including PM_2.5_, O_3_, and SO_2_ were controlled for. The estimated RR of stroke risk for each IQR increase (26 μg/m^3^) in NO_2_ was 1.0179 (95% CI, 1.0054 to 1.0306). This finding was in agreement with that of Tsai et al. demonstrating an association between NO_2_ and ischemic stroke admissions in Taiwan province [15]. The observed magnitude of elevated risk of ischemic stroke due to NO_2_ exposure in our study was smaller than the figure they reported [15]. Epidemiological evidence in Shanghai, China also displayed elevated risk of ischemic stroke mortality associated with the increase in daily concentration of NO_2_ [16]. In addition, a short-term evaluation of air pollution on stroke hospital admissions in the city of Wuhan, China found that exposure to NO_2_ is significantly associated with stroke hospitalizations during the cold season [23]. According to a recent systematic evaluation of short term association between air pollution and stroke worldwide, admission to hospital for stroke or mortality from stroke was associated with an increase in concentrations of NO_2_ (RR 1.014 per 10 ppb, 1.009 to 1.019) [24]. For the same-day exposure to SO_2_, this study demonstrated significant association with admissions for ischemic stroke when controlling for other pollutants including PM_2.5_, O_3_, and NO_2_. The estimated RR of ischemic stroke admission was 1.0179 (95% CI, 1.0054 to 1.0306) per IQR increase in SO_2_ (10 μg/m^3^). The similar association between ischemic stroke hospitalizations and exposure to SO_2_ was also suggested in a previous study [25].

More recently, possible causal effects on cardiovascular mortality due to particle exposure has raised greater public concern. Several potential mechanisms have been proposed, such as increased levels of particulate associating with elevated plasma viscosity [26], elevated risk of raised heart rate [27], and changes in heart rate variability [28]. In fact, studies have reported an association between NO_2_ exposure and plasma fibrinogen [29,30]. The findings supported the hypothesis that hemodynamic disturbances may lead to an increased risk of stroke [31]. We observed stronger short-term effects of NO_2_ and SO_2_ than PM_2.5_ on the risk of stroke hospital admissions in this study. In fact, gaseous pollutant from all kinds of vehicles has become one of the main atmospheric pollution sources in Guangzhou at present, and the ambient air quality standards are generally worse than those in the USA, Hong Kong, and the European Union [32]. This may partly explain why the effects of NO_2_ and SO_2_ were much higher than PM_2.5_ presented. The results of this study have implications in terms of public health. We estimated that there was a 3.45% and 1.12% increase in ischemic stroke risk associated with an IQR increase in the level of NO_2_ and SO_2_ that modeled as an averaged lag effects, respectively. The increased risks of ischemic stroke due to the exposures to NO_2_ and SO_2_ estimated in this work highlight the need for continued vigilance for the health risks of air pollution. In order to decrease the disease burden of stroke in the area, measures should be taken to increase public awareness about the ill effects of air pollution on population health, and educate the public about self-protection.

Several limitations of this study should be mentioned. First, this is a retrospective ecological study and the ecologic fallacy cannot be ruled out. The study participants were limited to community residents who had official residential records. Hence, a part of short-term floating workers in Guangzhou and patients outside the city might be excluded in study. Second, we assumed that the increase in hospital admissions resulted solely from an increase in disease occurrence. However, data in the hospital admission records usually induce biases due to some system-specific factors including clinical diagnosis on admission, admission policies, and reporting practices. Another thing to note here is that the assessment was limited to ambient air exposures at the community level, and did not include personal indoor air quality measurements or other indicators of individual exposures. The effects of noise pollution and occupational exposures may also have a potential impact on the magnitude of the observed associations here.

## 5. Conclusions

In conclusion, this study provided a thorough assessment about the associations between short-term exposures to ambient air pollutants and risk of ischemic strokes in Guangzhou, the most densely-populated city in south China. The findings will contribute to a better understanding of the effects of air pollution exposures on ischemic strokes, and to the planning of clinical services and emergency contingency response.

## Figures and Tables

**Table 1:** Basic characteristics of ischemic stroke patients by calendar year in Guangzhou during the time period of 2013–2015.

Characteristic	2013 (*n* = 27,532)	2014 (*n* = 32,751)	2015 (*n* = 35,279)
Gender, %			
Male	56.46	58.03	58.18
Female	43.54	41.97	41.82
Age, mean years	71.20	71.30	71.90
Age group, %			
<20	0.05	0.06	0.04
20–44	2.12	2.14	1.75
45–54	7.19	7.72	7.47
55–64	18.90	18.50	17.97
65–74	25.29	25.46	25.76
75 above	46.10	46.11	47.00
Missing	0.35	0.00	0.00
Ethnic group, %			
Han	99.68	99.29	98.67
Minority	0.26	0.58	0.26
Missing	0.06	0.13	1.07
Marital status, %			
Married	94.33	93.91	93.37
Divorced	0.35	0.41	0.43
Widowed	2.26	2.15	2.18
Single	1.02	1.03	1.14
Others	1.50	1.70	0.79
Missing	0.54	0.80	2.09
Length of hospital stay, median days	11.00	11.00	11.00
Hospital admission charge, median yuan	11,009.29	11,391.99	11,503.40

**Table 2:** Stroke risk ratios associated with an inter quartile range (IQR) increase in the levels of air pollutants including PM_2.5_, O_3_, SO_2_, and NO_2_.

Model	Pollutants	Lag 0 Model		Lag 1 Model	
		RR	95% CI	RR	95% CI
Single-pollutant model	PM_2.5_ only	1.0270 *	(1.0174, 1.0366)	1.0309 *	(1.0212, 1.0406)
	O_3_ only	1.0173 *	(1.0079, 1.0268)	1.0213 *	(1.0119, 1.0307)
	SO_2_ only	1.0363 *	(1.0262, 1.0465)	1.0317 *	(1.0216, 1.0419)
	NO_2_ only	1.0446 *	(1.0348, 1.0543)	1.0435 *	(1.0338, 1.0532)
Two-pollutant model	PM_2.5_ adjusted for O_3_	1.0238 *	(1.0133, 1.0345)	1.0263 *	(1.0157, 1.0370)
	O_3_ adjusted for PM_2.5_	1.0072	(0.9968, 1.0176)	1.0106 *	(1.0004, 1.0209)
	PM_2.5_ adjusted for SO_2_	1.0082	(0.9959, 1.0206)	1.0193 *	(1.0067, 1.0321)
	SO_2_ adjusted for PM_2.5_	1.0307 *	(1.0176, 1.0439)	1.0183 *	(1.0051, 1.0317)
	PM_2.5_ adjusted for NO_2_	0.9856	(0.9716, 0.9998)	0.9942	(0.9798, 1.0089)
	NO_2_ adjusted for PM_2.5_	1.0560 *	(1.0411, 1.0712)	1.0481 *	(1.0331, 1.0633)
	O_3_ adjusted for SO_2_	1.0052	(0.9952, 1.0154)	1.0119 *	(1.0019, 1.0220)
	SO_2_ adjusted for O_3_	1.0342 *	(1.0233, 1.0451)	1.0269 *	(1.0160, 1.0379)
	O_3_ adjusted for NO_2_	1.0046	(0.9949, 1.0144)	1.0110 *	(1.0014, 1.0207)
	NO_2_ adjusted for O_3_	1.0431 *	(1.0329, 1.0534)	1.0406 *	(1.0306, 1.0506)
	SO_2_ adjusted for NO_2_	1.0115	(0.9988, 1.0244)	1.0044	(0.9916, 1.0173)
	NO_2_ adjusted for SO_2_	1.0374 *	(1.025, 1.0500)	1.0408 *	(1.0283, 1.0534)
Multi-pollutant model	PM_2.5_ adjusted for O_3_, SO_2_ and NO_2_	0.9768 *	(0.9617, 0.9922)	0.9858	(0.9700, 1.0019)
	O_3_ adjusted for PM_2.5_, SO_2_ and NO_2_	1.0066	(0.9962, 1.0172)	1.0136 *	(1.0031, 1.0241)
	SO_2_ adjusted for PM_2.5_, O_3_ and NO_2_	1.0161 *	(1.0024, 1.0300)	1.0035	(0.9896, 1.0174)
	NO_2_ adjusted for PM_2.5_, O_3_ and SO_2_	1.0509 *	(1.0353, 1.0668)	1.0490 *	(1.0332, 1.0651

* *p* < 0.05, RR = relative risk, CI = confidence interval, IQR = interquartile range.
